# Investigation of Biomarkers Secreted from Adipose Tissue in Cattle Infected with *Theileria annulata*

**DOI:** 10.3390/vetsci13090911

**Published:** 2026-09-04

**Authors:** Cennet Nur Ünal, Murat Uztimür, Münir Aktaş, Mehmet Can Uluçeşme, Sümeyye Babacan, Mustafa İssi

**Affiliations:** 1 Department of Internal Medicine, Faculty of Veterinary Medicine, Bingöl University, 12000 Bingöl, Türkiye; muztimur@bingol.edu.tr; 2 Department of Parasitology, Faculty of Veterinary Medicine, Fırat University, 23119 Elazığ, Türkiye; maktas@firat.edu.tr (M.A.); mculucesme@firat.edu.tr (M.C.U.); 3 Department of Internal Medicine, Faculty of Veterinary Medicine, Fırat University, 23119 Elazığ, Türkiye; sbabacan@firat.edu.tr (S.B.); missi@firat.edu.tr (M.İ.)

**Keywords:** *Theileria annulata*, cattle, anemia, adipose tissue

## Abstract

*Theileria annulata* (*T. annulata*) is a parasite that causes tropical theileriosis, affecting around 250 million cattle in regions such as Turkey, Southern Europe, Northern Africa, and Asia. Anemia, a primary symptom of *T. annulata* infection, is critical to understanding the disease’s pathogenesis. Adipokines secreted from adipose tissue serve as signaling molecules influencing various systems, including energy metabolism and coagulation. This study aims to investigate the role of adipose tissue secretions (adiponectin, leptin, resistin, omentin-1) in the pathogenesis of the disease by evaluating the symptoms of anemia and inflammation associated with tropical theileriosis. The study involved 40 cattle, including 32 infected with *T. annulata* and eight healthy cattle. Hematological tests and measurements of serum adiponectin, leptin, resistin, and omentin-1 levels were conducted using ELISA kits. Adiponectin (*p* = 0.002) and omentin-1 (*p* = 0.001) levels were significantly higher in the infected groups compared to the control group. However, no significant differences were found in leptin and resistin levels between the infected and control groups. Adiponectin exhibited a moderate negative correlation with HCT (*p* = 0.011, r = −0.397) and RBC (*p* = 0.033, r = −0.338). A moderate negative correlation was found between HCT concentration and omentin-1 (*p* = 0.039, r = −0.328). The findings of this study suggest that the levels of adiponectin and omentin-1 are associated with *T. annulata* infection in cattle and that these adipokines may play a potential role in the pathogenesis of tropical theileriosis. These biomarkers may also have potential diagnostic value in the diagnosis of *T. annulata* infection.

## Simple Summary

Adipose tissue has generally been studied in veterinary medicine for its known roles in lipomobilization, metabolism, and insulin resistance. However, in human medicine, it has been demonstrated that adipose tissue regulates appetite, controls glucose and lipid metabolism, modulates the inflammatory response, influences energy metabolism, has neuroendocrine functions, plays a role in mechanisms associated with anemia, secretes hormones that interact with different systems, contributes in the pathogenesis of various diseases, and has therapeutic effects. Tropical theileriosis is commonly observed throughout Turkey and leads to high morbidity and mortality. In this disease, the multiplication of the pathogen in the lymph nodes, its spread throughout the body via the lymphatic circulation, and the occurrence of a multiplication cycle in erythrocytes cause anemia. Despite anemia being one of the most important symptoms of the disease, its exact mechanism has not yet been fully elucidated. For this reason, the project investigated the role of adipose tissue secretions (adiponectin, leptin, resistin, omentin-1) in the pathogenesis of the disease by evaluating the symptoms of anemia and inflammation that occur in tropical theileriosis. This study proposes a new mechanism involved in the diagnosis and pathogenesis of tropical theileriosis in veterinary medicine, and also contributes to a better understanding of the role of adipose tissue in the course of diseases.

## 1. Introduction

*Theileria annulata* (*T. annulata*) is a parasite that causes tropical theileriosis and is transmitted by ticks. It has been reported that approximately 250 million cattle are at risk of *T. annulata* infection in regions where the disease is endemic, including Turkey, Southern Europe, North Africa, and Asia [1,2,3]. In cattle, the disease develops when sporozoites enter the host’s body during blood feeding by an infected tick [4]. Clinical signs in naturally infected animals most commonly include anemia, elevated body temperature, enlarged lymph nodes, anorexia, weakness, respiratory distress, petechial hemorrhages in the conjunctiva, oral and nasal mucosa, and corneal opacification [5,6,7].

Adipose tissue plays a central role in regulating whole body energy and glucose homeostasis through its functions at both the organ and systemic levels. It also interacts with other organs and systems at both local (autocrine/paracrine) and systemic (endocrine) levels through different mediators and active compounds known as adipokines [8]. Through these interactions, adipose tissue is considered an organ with the potential to regulate numerous biological processes, including the coagulation mechanism, anemia, neuroendocrine function, and inflammation [9,10,11]. It is stated that adiponectin affects the proliferation and function of hematopoietic stem cells, and that leptin and its receptor play a role in the proliferation and differentiation of hematopoietic progenitor cells, including erythropoietic pathways [12,13]. Studies investigating the role of adipokines in protozoal infections in veterinary medicine are limited. However, in dogs naturally infected with *Leishmania infantum*, Di Loria et al. [14] showed that leptin levels increased, while Muñoz-Prieto et al. [15] demonstrated that adiponectin levels decreased. Therefore, it is thought that adipokines may be affected in protozoal infections.

Studies in humans [16,17] have identified a relationship between adipose tissue and diseases associated with anemia and have shown that adipose tissue plays significant roles in various diseases affecting cattle [18,19]. However, no studies investigating the adipose tissue hormones of cattle infected with *T. annulata*. In the presented study, the aim is to evaluate adipose tissue hormones (adiponectin, leptin, resistin, and omentin-1) in cattle infected with *T. annulata* and to investigate the relationships of these hormones with anemia and the disease.

## 2. Materials and Methods

### 2.1. Animal Selection

**Figure 1:**
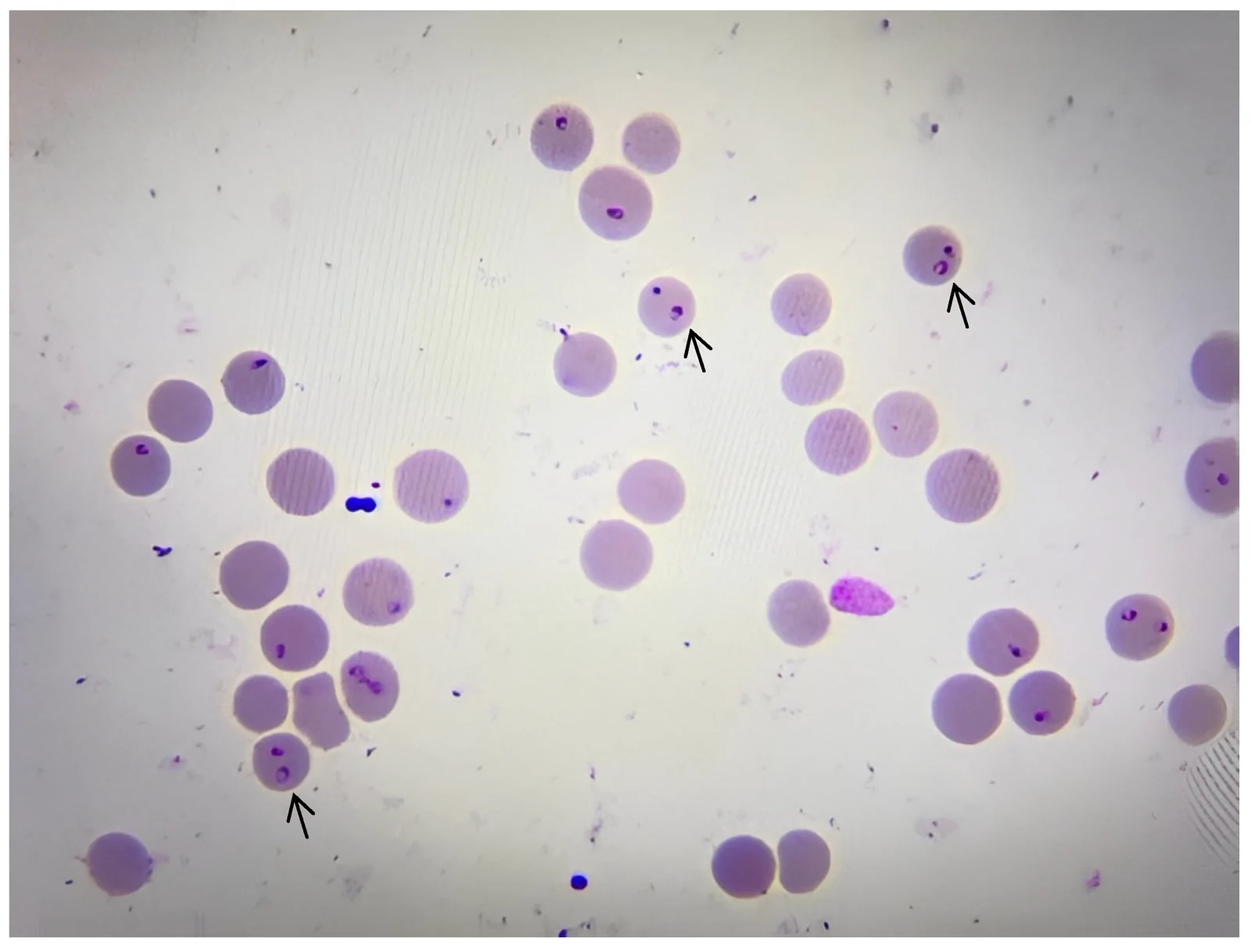
Representative intraerythrocytic piroplasms of *T. annulata* in a Giemsa-stained blood smear, ×100 oil-immersion objective, arrows: intraerythrocytic piroplasms.

Systematic clinical examinations were performed on the animals brought to the Department of Internal Medicine. Following systematic clinical examination [20], blood samples were collected from the peripheral ear capillaries of cattle [21] showing signs of theileriosis, such as increased body temperature, anemia, jaundice, hyperemia, unilateral or bilateral lymph node hypertrophy, and petechia on the conjunctiva and various parts of the body [22,23,24]. Thin blood smears were prepared from the blood samples taken on the slide and left to air dry. After the blood smears were fixed with methanol for 5 min, they were stained with Giemsa stain diluted to 8% with distilled water for approximately 30 min. After staining, the blood smears were washed and air-dried, and examined under a ×100 objective using immersion oil. In each blood smear, 50 microscopic fields were scanned, and preparations evaluated as negative were examined twice before confirming negativity [25]. The presence of agents in the form of needles, rings, ovals, rods, or commas within erythrocytes was considered positive for theileriosis [26]. The preparations were evaluated by an experienced researcher. Since it is known that the samples were obtained from clinically suspicious animals, the microscopic evaluation was not conducted in a blinded manner to the clinical condition. The image of *Theileria* spp. is shown in Figure 1.

### 2.2. DNA Isolation and Polymerase Chain Reaction (PCR)

Following clinical and microscopic examinations, 2 mL blood samples were collected from the jugular vein of animals diagnosed with tropical theileriosis and those in the control group, using EDTA tubes. Genomic DNA isolation was performed in the laboratory of the Department of Parasitology, Faculty of Veterinary Medicine, Fırat University. A commercial kit (Invitrogen, PureLink™ Genomic DNA Mini Kit, Thermo Fisher, Waltham, MA, USA) was used for genomic DNA isolation from 200 µL of blood. DNA isolation was performed according to the manufacture’s protocol, and the purified DNA was eluted in 100 µL of elution buffer. *Theileria annulata*-positive blood samples available in our laboratory stocks were used as controls for DNA isolation.

DNA samples obtained in this study were amplified for *T. annulata* by PCR. In PCR analysis, primers N516 (5′ GTAACCTTTAAAAACGT3′) and N517 (5′ GTTAC-GAACATGGGTTTT3′), which amplify 721 bp of the 30 kDa merozoid surface antigen gene of *T. annulata*, developed by d’Oliveira et al. [27] were used. The PCR heat conditions used in the amplification of *T. annulata* are given in Table 1, and the PCR content is given in Table 2. The PCR reagents were obtained from Thermo Scientific (Thermo Scientific, Thermo Fisher Scientific, Waltham, MA, USA) and amplification was performed using a thermal cycler (SensoQuest, Labcycler Gradient, Göttingen, Germany). Amplification was performed using a thermal cycler (SensoQuest, Labcycler Gradient, Göttingen, Germany).

Positive and negative DNA controls were included in each PCR assay. The reference positive control DNA, *T. annulata* (EU622823), was obtained from our laboratory stocks and had previously been confirmed by DNA sequence analysis. As a negative DNA control, a calf DNA sample, also available in our laboratory stocks and previously tested negative for *Theileria*, *Babesia*, and *Anaplasma* species, was used. A no-template control (NTC) and a negative extraction control were not included in the assays.

All of the amplicons (5 µL) obtained from PCR analysis were electrophoresed on 1.4% agarose gels containing ethidium bromide in 1× TAE buffer at 100 V for 1 h. DNA fragments were visualized under UV illumination using a Quantum Vilber Lourmat gel imaging system (Marne-la-Vallée, France). A 100 bp DNA ladder (GeneDireX, Miaoli County, Taiwan) was used as a molecular-weight marker for fragment-size estimation.

### 2.3. Classification of Animals Used in Research

Samples were taken from animals that presented to the clinic and satisfy the study criteria. The animals were not pre-selected based on hematocrit values during sampling; they were assigned to the relevant study groups based on hematocrit values after the completion of sampling and clinical/laboratory evaluations. To ensure equal sample sizes across groups, eight animals were assigned to each group. Since Group 3 contained a total of 12 animals meeting the study criteria, 8 were randomly selected from this pool, while the remaining four were excluded from the final analysis. In the control group, the eight animals meeting the criteria were selected directly, without any excess sampling; all of these animals were included in the study. Consequently, no pre-selection based on HCT values was performed during sampling; however, as the study aimed to group animals according to anemia severity at the outset, group sizes were balanced following the completion of sampling and clinical/laboratory evaluations.

PCR analysis was performed on samples taken from animals whose clinical and laboratory findings were consistent with the study criteria to confirm the infection. The PCR analysis results of four animals were negative, so these animals were excluded from the study and not included in the final evaluation.

The animals’ previous treatment histories varied. Some animals had received treatment for different diseases long before being included in the study, while some animals had no treatment history at all. Previous treatment history was recorded during the clinical evaluation. The animals were evaluated through systematic clinical examination and laboratory analyzes for accompanying diseases and possible co-infections, and those with other diseases or co-infections were not included in the study. Theileriosis diagnosis was confirmed by clinical and laboratory findings as well as PCR analysis. Four animals that tested negative in the PCR analysis were removed from the study and excluded from the final evaluation. It was reported by the animal owners that the animals’ diet included hay and concentrate feed, and they had access to water ad libitum. Simmental and Holstein breed animals were used in the study.

A total of 40 cattle were used in this study. Of these, 32 animals were confirmed to be infected with *T. annulata* through hematological, microscopic, and PCR methods. The remaining eight animals were determined to be completely healthy based clinical examination, absence of ticks, hematological evaluation, parasitological examination, and PCR control group. The control group animals were selected based on specific criteria to ensure comparability with the infected group. These criteria included factors such as age, physiological condition, body condition score (average 2.5–3.5), and environmental conditions. This approach was taken to increase statistical validity by reducing physiological or environmental differences between the experimental and control groups, aside from the infection status. Animals infected with *T. annulata* were grouped according to the severity of anemia based on their hematocrit values as follows. Later, the parasitemia rate of the animals included in the study was determined according to the method described by Rezaei and Dalir-Naghadeh [28], and the degree of tick infestation was determined as specified by Castañeda Arriola et al. [29]. The parasitemia rate was evaluated by counting the infected erythrocytes within 100 erythrocytes in the microscopic examination of blood smears, with the tick infestation degree classified as low (0–30 ticks), moderate (31–60 ticks), and high (≥61 ticks).

Group 1: Cattle infected with *T. annulata* without anemia (≥24%, n = 8)

Group 2: Cattle infected with mildly anemic *T. annulata* (20–23.99%, n = 8)

Group 3: Cattle infected with moderately anemic *T. annulata* (12–19.99%, n = 8)

Group 4: Cattle infected with severely anemic *T. annulata* (≤11.99%, n = 8) [30].

Animals with similar physical characteristics (breed, age, sex, body condition score) and animals that had been previously treated for *Theileria* spp., had a different disease identified in clinical and laboratory examinations, and had mixed infection were not included in the study. Cattle in the control group were included if they were determined to be negative by PCR analysis. The age, sex, and breed characteristics of the animals are shown in Table 3.

### 2.4. Sample Collection Protocol, Hematology and Elisa Analysis

From the cattle that underwent PCR analyses and were grouped, 3 mL blood samples were taken from the jugular vein into EDTA tubes for hematological examinations, and 10 mL blood samples were taken into gel serum tubes for adipose tissue biomarker analyses.

Hematological examinations [total leukocyte count (WBC), hematocrit value (HCT), erythrocyte count (RBC), mean corpuscular volume (MCV), hemoglobin amount (HGB), platelet count (PLT), mean corpuscular hemoglobin concentration (MCHC), mean corpuscular hemoglobin amount (MCH)] were determined from blood samples taken in EDTA tubes immediately after collection using an automatic blood counting device (BC-5000Vet Shenzhen Mindray Bio-Medical Electronics Co., Ltd., Shenzhen, China).

Serum was obtained from blood samples taken in gel tubes [24]. Blood samples were centrifuged at 7000 rpm for 5 min within a maximum of 3 h after collection, and the obtained serums were stored at −20 °C for a maximum of 6 months until analysis. Serum samples were not subjected to repeated freeze–thaw cycles. Adiponectin (Coonkoon, CK-BIO-18391, Shanghai, China), leptin (Coonkoon, CK-BIO-18571, China), resistin (Coonkoon, CK-BIO-18648, China), and omentin-1 (Coonkoon, CK-BIO-24595, China) levels were measured using bovine-specific ELISA kits. Samples were also not subjected to pre-dilution; according to the manufacturer’s protocol, 10 µL of serum and 40 µL of sample diluent were added to each well and analyzed in a single measurement. Absorbance was measured at 450 nm using a microplate reader (BioTek ELx800, Winooski, VT, USA). The intra-assay variation coefficient of the ELISA kits used in the study was <7%, and the inter-assay variation coefficient was <10%. The assay ranges were 1–80 ng/mL for adiponectin, 1–40 ng/mL for leptin, 5–100 ng/mL for resistin, and 1–64 ng/mL for omentin-1. The analytical sensitivity values are 0, 0.1, 1.0, and 0.1 ng/mL, respectively. The relevant values are provided by the manufacturer in the ELISA kit data sheet. The results of all samples remained within the relevant analytical measurement ranges. ELISA analyses on the sera were performed as single measurements. The personnel conducting the ELISA analyzes knew that the samples belonged to either the patient or control animals, but they were blinded to the subgroup distribution of the patient animals.

### 2.5. Statistical Analysis

Sample size was calculated using the G*Power 3.1 program, assuming that comparisons between groups would be made using one-way analysis of variance (ANOVA). Effect size was determined as 0.65, alpha error level as 0.05, and statistical power (1–β) as 85% [31]. Based on these parameters, a total of 40 animals, approximately eight individuals for each group, were included in the study. The effect size of 0.65 was not taken from a previous study or pilot data. It was determined as a priori assumption regarding the expected magnitude of the difference between the groups before the study.

SPSS statistical program (IBM SPSS Version 22.0) was used for data analysis (IBM SPSS, 2013). Data were first examined with a normality test (Shapiro–Wilk test). The assessment of normality is based on the evaluation of both statistical test results and Q-Q plots together.

ANOVA was performed for data for leptin, resistin, and omentin-1, which showed normal distribution, and the Levene test was applied to check the equality of variances. Then, post hoc LSD/Tamhane was used. The Kruskal–Wallis test was used for adiponectin and all hematological data, and the Mann–Whitney U test was preferred for pairwise comparisons between groups. In cases where a statistical difference was found in one-way ANOVA, LSD was used when homogeneity of variance was achieved, and the Tamhane test was used when homogeneity could not be achieved. When a significant difference was detected in the Kruskal–Wallis test, a Bonferroni correction was applied to adjust the Type I error rate that could occur in the Mann–Whitney U test.

Receiver Operating Characteristic (ROC) analysis was used to determine the area under the curve (AUC), sensitivity, specificity, and cut-off values of the parameters between patient and healthy groups without discrimination according to the severity of anemia. To evaluate the performance of adiponectin, resistin, and omentin-1 in distinguishing diseased animals from control animals, ROC analyses were conducted as exploratory analyses. In the ROC analysis, 32 *T. annulata* infected and 8 control animals were evaluated. Cut-off values were selected by considering the balance between sensitivity and specificity based on the ROC curve coordinates. The results of the ROC analyses were reported along with AUC values and their corresponding 95% confidence intervals, cut-off values, sensitivity, specificity, and the number of true positive/true negative animals. AUC values: 0.500, non-diagnostic; 0.600–0.700, poor diagnostic performance; 0.700–0.800 was considered acceptable diagnostic performance; 0.800–0.900 was considered good diagnostic performance, and above 0.900 was considered very good diagnostic performance [32]. Correlation between variables was determined by Spearman rank correlation analysis as described by Little and Hills [33] Correlation strength was interpreted based on the absolute value of the correlation coefficient (|r|), with |r| < 0.30 considered low, 0.30 ≤ |r| < 0.60 considered moderate, and |r| ≥ 0.60 considered high.

## 3. Results

### 3.1. Clinical and Microscopic Examination Findings

Parasitemia, tick infestation status, and clinical examination findings of cattle infected with *T. annulata* grouped based on hematocrit values are shown in Table 4.

### 3.2. Hematological Analysis Findings

Hematological results in the *T. annulata* infected and control groups are given in Table 5. For Group 4, HGB and HCT values were verified against the analyzer measurements, and it was observed that the obtained values were identical to those reported in the article. However, the observed variations may not be entirely attributable to biological variation. Therefore, the results should be interpreted with caution.

### 3.3. Elisa Analysis Findings

The results of the ELISA analyses conducted on the *T. annulata* infected and control groups are presented in Table 6.

### 3.4. Roc Analysis Findings

**Figure 2:**
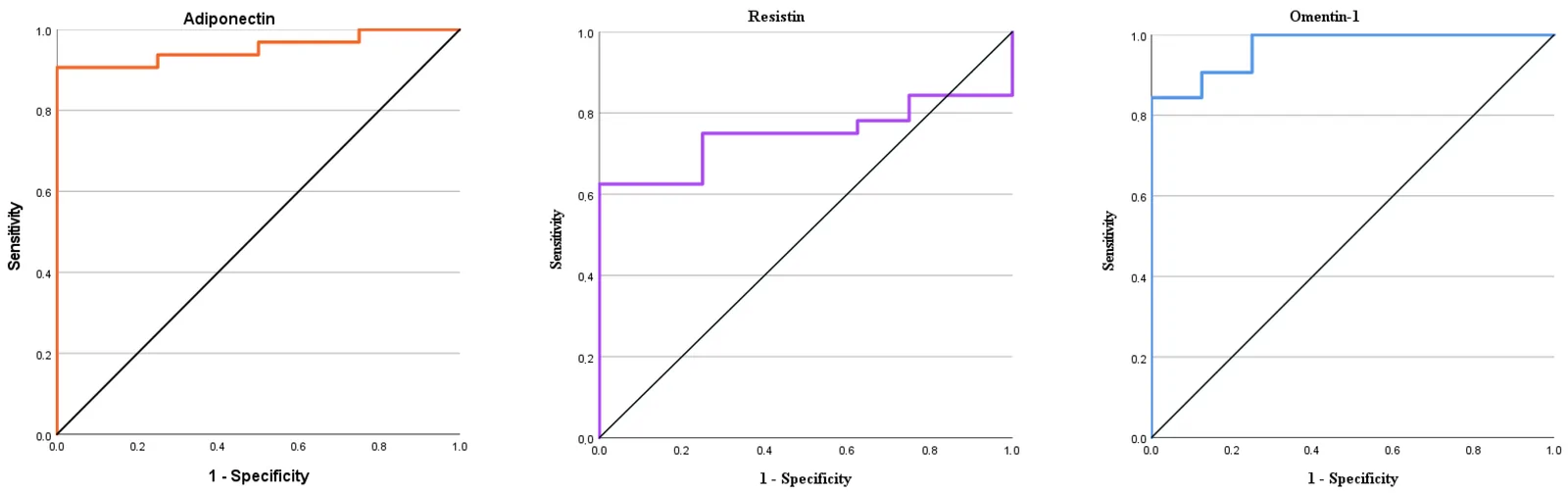
Graphical representation of ROC analysis results of adiponectin, resistin, and omentin-1 in cattle infected with *T. annulata*.

It was determined that adiponectin exhibited very good diagnostic performance with an AUC of 0.953, a cut-off value of 16.43 ng/mL, 90% sensitivity, and 87% specificity. Resistin was observed to have an acceptable diagnostic performance with an AUC of 0.746, a cut-off value of 23.26 ng/mL, 75% sensitivity, and 75% specificity. It was determined that omentin-1 showed very good diagnostic characteristics with an AUC of 0.969, a cut-off value of 12.97 ng/mL, 87% sensitivity, and 87% specificity. The ROC analysis results of adipose tissue hormones are presented in Table 7 and Figure 2.

### 3.5. Correlation Analysis Findings

The correlations between HCT, HGB, RBC, and adipose tissue hormones among all cattle included in the study are presented in Table 8. Spearman’s correlation analysis indicated that HCT had a high positive correlation with HGB (*p* = 0.001, r = 0.733) and RBC (*p* = 0.001, r = 0.863), and moderate negative correlations with adiponectin (*p* = 0.011, r = −0.397), resistin (*p* = 0.007, r = −0.421), and omentin-1 (*p* = 0.039, r = −0.328). RBC showed a moderate negative correlation with adiponectin (*p* = 0.033, r = −0.338).

### 3.6. PCR Analysis Results

**Figure 3:**
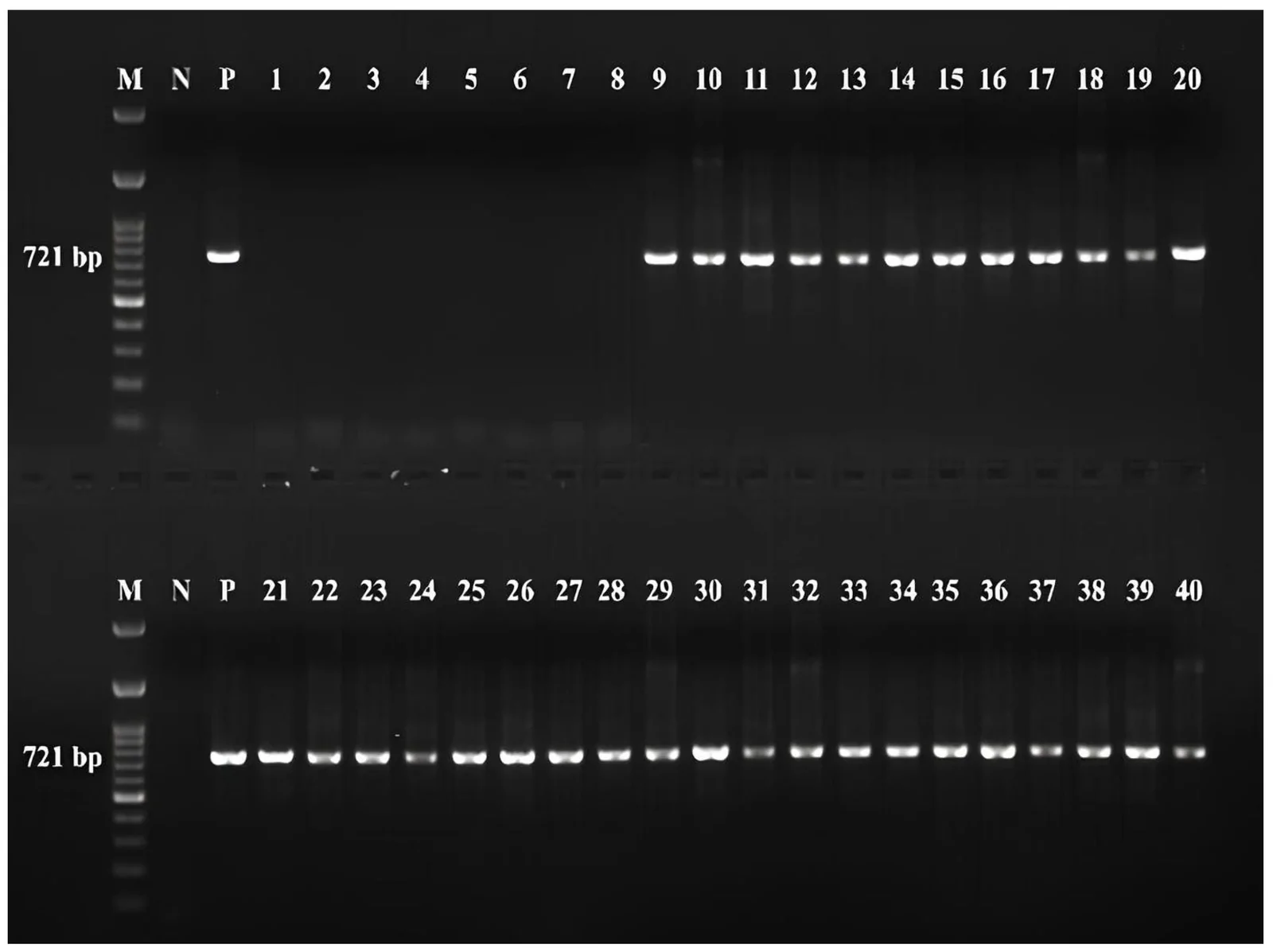
Gel image of PCR amplification products of *T. annulata* obtained with N516/N517 primers. M, 100 bp marker; N, negative control (N, DNA sample from a calf negative for *Theileria*, *Babesia*, *Anaplasma* species); P, positive control (*T. annulata*-EU622823). 1–8 control group, 9–16 group 1, 17–24 group 2, 25–32 group 3, 33–40 group 4.

In the study, both microscopic blood smears and molecular PCR analysis were used together to confirm *T. annulata* infection. PCR results were compared with microscopic diagnosis. The obtained data showed that the microscopic method was reliable, especially at moderate and high parasitemia levels. Therefore, not only microscopically positive animals but also all cases confirmed positive by PCR were included in the study. All animals in the experimental groups were found to be positive for the *T. annulata* agent through PCR analysis, while animals in the control group were found to be negative for *T. annulata*. These results are presented in Figure 3.

## 4. Discussion

Tropical theileriosis is an important protozoan disease caused by *T. annulata*, commonly observed in cattle and associated with high morbidity and mortality [34]. Although some pathogenesis of the anemia occurring in the disease has been elucidated, there is still a need to investigate different mechanisms [35]. Adipose tissue contributes to the pathogenesis of various diseases due to its multiple functions [8]. This study investigates the function of adipose tissue hormones in the development of *T. annulata* infection in cattle.

Anemia is one of the most important symptoms of tropical theileriosis [36,37]; its exact mechanism remains unclear [35,38,39]. Several factors contribute to anemia development, including the immune response [40], the formation of extravascular hemolytic anemia [35,38,41], increased osmotic fragility of erythrocytes [42], and the elimination of damaged erythrocytes by macrophages in the spleen, lymph nodes, and other organs of the reticuloendothelial system. It is stated that different conditions, such as disrupted metabolic processes [43,44], lipid peroxidation, and oxidative injury [28], play a role in the pathogenesis of anemia. The RBC, HCT, and HGB concentrations observed in this study are consistent with anemia findings reported in previous studies. Similar hematological parameters have been documented by Hasanpour et al. [44] who reported RBC 4.4 × 10^6^/µL and HCT 21.7%, Omer et al. [35] who found RBC 3.96 × 10^12^/L, HGB 7.17 g/dL, and HCT 21.8%, and Issi et al. [45] who observed RBC 4.06 × 10^6^/mm^3^ and HGB 6.74 g/dL. These findings support the association between tropical theileriosis and anemia development. Statistically, there is no significant difference in MCHC levels between the groups, but the MCHC, which is inconsistent with the HGB and HCT values in group 4, should be interpreted with caution. Although the analyzes were conducted with an automatic hematology device, they may still affect the relevant values.

In human medicine, adiponectin has been shown to play an important role in the pathogenesis of disease in cases of anemia. Kim et al. [46] identified elevated blood adiponectin levels in patients without anemia at baseline as a significant risk factor for the later onset of anemia and illustrated its potential role as an independent predictor of the condition. A positive correlation has been shown between anemia and adiponectin, and it is stated that adiponectin levels can be used as a predictor for anemia [47]. Additionally, a negative correlation has been found between adiponectin and erythrocyte count, hemoglobin amount, and hematocrit value [48]. Previous studies suggest that adiponectin may be associated with the pathogenesis of anemia through its potential effects on hematopoietic processes, independent of renal function [17,48,49]. In this study, although adiponectin did not differ between infected groups, it was found to be significantly higher in all groups compared to the control group (*p* = 0.002). Adiponectin exhibited a moderate negative correlation with HCT (*p* = 0.011, r = −0.397) and RBC (*p* = 0.033, r = −0.338) parameters, which are key indicators for evaluating the severity of anemia. In addition, it was determined that adiponectin could help in the diagnosis of the disease with 90% sensitivity and 87% specificity at levels above the cut-off value of 16.43 ng/mL with an AUC value of 0.953.

Leptin, while widely recognized for its roles in cattle [19,50], has also been shown to play significant functions in anemia and inflammation related conditions in humans and mice [16,51]. Togo et al. [52] found a negative correlation between hemoglobin and leptin levels. It is stated that leptin and its receptor B219, which are expressed in hematopoietic cells, can form a hematopoietic pathway that affects erythropoiesis. In addition, it has been shown that leptin and erythropoietin work synergistically to enhance erythrocyte development in anemia [12]. In a study by Dedoussis et al. [16], a negative correlation was found between leptin levels and soluble transferrin receptor levels, which are associated with anemia in patients with β-thalassemia. The course of leptin was attributed to the energy metabolism disorder caused by the disease and the hormone’s role in hematopoiesis. In this study, although leptin levels were higher in *T. annulata* infected groups compared to the control group, no statistically significant difference was found (*p* = 0.062). The results obtained for cattle for leptin were higher than those reported by Guzel and Tanrıverdi [53], Eğritağ et al. [19], and Sabzikar et al. [54], and similar to those reported by Duru et al. [55]. The differences observed between the leptin concentrations and statistical analysis results reported in this study and those in previous studies may be attributed to the study population, disease status, and disease mechanism. Furthermore, these discrepancies in the results are thought to stem from physiological changes such as nutrition, adipose tissue status, pregnancy, and lactation as well as variations in measurement techniques and analytical methods.

Research on resistin in cattle generally focuses on its functions associated with energy and lipid metabolism [56]. In cattle, plasma resistin levels vary between 21–100 ng/mL during pregnancy and early, and late lactation stages. These levels have been observed to increase one week after calving, and recombinant resistin has been shown to stimulate lipolysis in vitro [56,57]. However, studies conducted in humans have shown a connection to many diseases, such as atherosclerosis, cardiovascular disease, cancer, asthma, inflammatory bowel diseases, and chronic kidney disease [58]. In β-thalassemia patients characterized by inflammation and anemia, significantly higher levels were found compared to the control group, with a positive correlation with ferritin and a negative correlation with hemoglobin concentration. This difference was attributed to the hormone’s role in the inflammation process [59]. The resistin level obtained in this study was within the range reported by Reverchon et al. [57] and Weber et al. [56]. However, no statistically significant difference was found between the *T. annulata* and control groups (*p* = 0.122). The obtained results did not show any variation between the patient and control groups, contrary to what Zhang et al. [60] and Dawoud et al. [59] reported. The reason for this situation can be explained by factors such as differences in the study population and disease pathogenesis, environmental conditions, and analysis methods. However, the moderate negative correlation between resistin levels and HCT (*p* = 0.007, r = −0.421) and the acceptable diagnostic performance in the ROC analysis (0.746 AUC, 23.26 ng/mL cut-off value, 75% sensitivity, 75% specificity) suggest that resistin may have potential importance in disease-related biological processes and diagnostic evaluation. However, due to the lack of significant differences between the groups, the ROC findings regarding resistin should be interpreted with caution.

*T. annulata* infection and anemia are conditions that trigger strong inflammatory responses in the immune system [22]. Omentin-1 is known as a hormone that regulates the relationship between metabolism and inflammation [61,62]. However, studies in human medicine have revealed different roles of omentin-1, like other adipose tissue hormones, in various disease processes [62]. Yin et al. [63] showed that omentin-1 levels were decreased in people with inflammatory bowel disease, and by Qi et al. [61] that it was decreased in people with respiratory distress syndrome and could be used therapeutically. Zabetian-Targhi et al. [64] found that omentin was negatively correlated with IL-6 and TNF-α concentrations and positively correlated with IL-13, IL-4, and IL-1β in obese humans. Zhong et al. [65] reported a decrease in patients with acute coronary syndrome compared to healthy controls. However, there is a limited number of studies evaluating the role of omentin-1 in different diseases in cattle. In a study conducted in cattle in the transition period [66], it was reported that omentin-1 was positively correlated with markers of energy metabolism, and this hormone may be an important adipokine for energy management. There is no study evaluating omentin-1 levels in cattle in diseases characterized by inflammation and anemia. Therefore, there is no data to discuss the findings. In this study, although no significant difference was observed in omentin-1 levels between the *T. annulata* infected groups, it was significantly higher in all infected groups compared to the control group (*p* = 0.001). A moderate negative correlation was found between the HCT concentration evaluated in relation to anemia and omentin-1 (*p* = 0.039, r = −0.328). Additionally, it was determined that the ROC analysis showed very good diagnostic characteristics with an AUC of 0.969, a cut-off value of 12.97 ng/mL, 87% sensitivity, and 87% specificity. In this context, the increase in omentin-1 levels in *T. annulata* infected animals can be considered an adaptive response to various mechanisms affected in the pathogenesis of the disease in the organism.

One limitation of this study is that biomarkers of the inflammatory response and oxidative damage caused by the infection, such as CRP, haptoglobin, ferritin, malondialdehyde, and total antioxidant capacity, were not analyzed. Nevertheless, since these markers have previously been investigated in animals infected with *T. annulata*, our findings are supported by the existing literature. However, because these specific analyses were not performed in the current study population, they cannot be directly correlated with the findings of the present study. Another limitation of the study is the distribution of sex and breed among the animals. These characteristics should be regarded as potential factors that could alter hormone secretion from adipose tissue. The correlation analysis aimed to evaluate differences among all groups; however, because only the infected groups were included, the findings should be interpreted with caution. A combined assessment of these parameters could provide deeper insight into the pathogenesis of *T. annulata* infection and, by correlating them with hematological findings, allow for a more comprehensive characterization of the systemic effects of the disease. Although species identification in the present study was based on a reference positive control previously validated by species-specific PCR and DNA sequence analysis with established primers, the clinical amplicons themselves were not sequenced. Sequencing of the clinical amplicons would have further strengthened species-level verification and should therefore be considered a limitation of the present study. Furthermore, the absence of a no-template control (NTC) and a negative extraction control represents an additional limitation. The fact that serum samples were analyzed by ELISA as single measurements rather than in duplicate is also a limitation. The current findings require validation in larger, independent animal populations. Future studies would benefit from the simultaneous evaluation of markers of inflammation, oxidative stress, and adipose tissue.

## 5. Conclusions

In conclusion, the levels of adiponectin, leptin, resistin, and omentin-1 were evaluated in cattle infected with *T. annulata*. Adiponectin and omentin-1 levels were higher in all infected groups than in the control group. These findings suggest that changes in adiponectin and omentin-1 levels may be related to disease-associated biological processes and could play a role in the pathogenesis of tropical theileriosis. However, the causal nature of these relationships cannot be determined from the current cross-sectional study. Additionally, adiponectin and omentin-1 may serve as potential biomarkers for the diagnosis of *T. annulata* infection. However, no independent internal or external validation was performed for the ROC analysis, and this should be considered a limitation of the study. Further studies are needed to clarify the biological significance of these adipokines in tropical theileriosis.

## Figures and Tables

**Table 1:** PCR temperature conditions used in *T. annulata* DNA amplification.

Operation	Temperature (°C)—Time (Minutes)	Number of Cycles
Predenaturation	94—5	1
Denaturation	94—1	40
Annealing	56—1	
Extension	72—1	
Last extension	72—10	1
Hold	4 °C	

**Table 2:** PCR reaction content.

Substance	Amount (μL)	Concentration	Final Concentration
Sterile distilled water	13		
PCR buffer	2.5	10×	1×
MgCl	2.5	25 mM	2.5 mM
dNTP	2	1.25 mM	0.10 mM
Taq DNA polymerase	0.1	5 U/µL	0.5 U
Revers primer	1.25	20 pmol/µL	1 pmol/µL
Forward primer	1.25	20 pmol/µL	1 pmol/µL
Template DNA	2.5		
Final reaction volume: 25 μL			

**Table 3:** The age, sex, and breed characteristics of animals.

Parameters	Group 1 (n = 8)	Group 2 (n = 8)	Group 3 (n = 8)	Group 4 (n = 8)	Control Group (n = 8)
Age (day), median	650.00	725.00	650.00	950.00	900.00
Sex, n (%)					
Male	3 (37.5%)	2 (25.0%)	3 (37.5%)	1 (12.5%)	0 (0%)
Female	5 (62.5%)	6 (75.0%)	5 (62.5%)	7 (87.5%)	8 (100%)
Breed, n (%)					
Holstein	4 (50.0%)	5 (62.5%)	0 (0%)	2 (25.0%)	6 (75.0%)
Simmental	4 (50.0%)	3 (37.5%)	8 (100%)	6 (75.0%)	2 (25.0%)

n: number of animals.

**Table 4:** Parasitemia, tick infestation status and clinical examination findings of cattle infected with *T. annulata* grouped according to hematocrit value.

Groups	Hematocrit Value (%)	Level of Parasitemia (%)	Degree of Tick Infestation	Clinical Findings
Group 1	≥24%	6.00% (4.00–8.00%)	Low	Increased body temperature to 38.5, mild symptoms such as fatigue, or no clinical symptoms were observed.
Group 2	20–23.99%	4.00% (2.00–6.00%)	Low	Enlargement of lymph nodes, mild anemia, increased body temperature.
Group 3	12–19.99%	13.00% (7.00–15.00%)	Low	There was mild anemia, increased body temperature, pale mucous membranes, enlarged lymph nodes, and weakness.
Group 4	≤11.99%	8.50% (6.00–10.00%)	Low	Increase in body temperature, marked pallor and/or petechiae on mucous membranes, and pronounced weakness.

Data are presented as median % (minimum–maximum %).

**Table 5:** Hematological analysis results and statistical significance levels between *T. annulata* infected and control group cattle.

Parameters	Group 1 (n = 8)	Group 2 (n = 8)	Group 3 (n = 8)	Group 4 (n = 8)	Control Group (n = 8)	*p* Value
WBC (×10^9^/L)	5.95 (4.37–15.70)	8.65 (3.10–18.70)	5.45 (4.00–19.00)	8.40 (4.50–31.00)	11.25 (5.40–16.40)	0.455
HCT (%)	26.50 ^a^ (24.00–30.00)	21.00 ^b^ (20.00–23.00)	17.50 ^c^ (12.00–18.00)	8.50 ^d^ (5.00–10.00)	30.00 ^e^ (25.30–32.90)	<0.001
RBC (×10^12^/L)	6.78 ^a^ (4.72–8.26)	3.57 ^b^ (1.05–5.37)	3.22 ^b^ (2.48–6.21)	1.24 ^c^ (0.47–1.91)	7.14 ^a^ (6.04–7.50)	<0.001
MCV (fL)	45.05 (32.60–73.30)	49.35 (33.40–83.90)	35.65 (33.10–91.10)	44.50 (21.20–54.60)	48.25 (33.40–56.20)	0.762
HGB (g/dL)	10.35 ^a^ (7.60–12.70)	7.75 ^b^ (4.90–10.00)	5.30 ^b^ (2.80–8.20)	2.90 ^c^ (1.40–11.40)	9.95 ^a^ (8.30–11.10)	<0.001
PLT (×10^9^/L)	226.50 (160.00–281.00)	240.00 (150.00–282.00)	231.00 (124.00–360.00)	122.00 (75.00–442.00)	174.00 (138.00–350.00)	0.455
MCHC (g/dL)	42.55 (25.10–65.80)	57.75 (25.10–79.60)	49.95 (30.80–63.70)	49.35 (17.80–182.20)	41.20 (30.60–58.20)	0.406
MCH (pg)	15.40 (11.90–22.60)	19.30 (8.40–44.80)	18.85 (15.60–26.90)	14.30 (7.90–25.60)	15.40 (12.20–25.60)	0.160

n: number of animals, WBC: total leukocytes, HCT: hematocrit value, RBC: erythrocyte count, MCV: mean erythrocyte volume, HGB: hemoglobin concentration, PLT: platelet count, MCHC: mean corpuscular hemoglobin concentration, MCH: mean corpuscular hemoglobin. ^a^, ^b^, ^c^, ^d^, ^e^: The difference between measurements carrying different letters in the same row is statistically significant. The data is provided as median and minimum–maximum values.

**Table 6:** Adipose tissue hormone concentrations and statistical significance levels between the *T. annulata* infected group and the control group.

Parameters	Group 1 (n = 8)	Group 2 (n = 8)	Group 3 (n = 8)	Group 4 (n = 8)	Control Group (n = 8)	*p* Value
Adiponectin (ng/mL)	18.62 ^a^ (17.24–20.40)	19.22 ^a^ (15.35–22.41)	19.09 ^a^ (17.60–23.76)	18.71 ^a^ (13.95–26.85)	14.48 ^b^ (11.69–16.81)	0.002
Leptin (ng/mL)	10.49 (9.31–12.30)	10.82 (9.69–14.90)	10.56 (9.07–12.39)	9.57 (8.13–12.84)	9.40 (7.56–10.70)	0.062
Resistin (ng/mL)	23.32 (19.42–29.04)	22.88 (17.43–31.83)	24.88 (23.42–30.04)	26.39 (21.42–29.25)	23.02 (20.76–23.74)	0.122
Omentin-1 (ng/mL)	15.47 ^a^ (12.55–17.28)	15.75 ^a^ (12.31–18.43)	14.80 ^a^ (12.68–16.97)	14.30 ^a^ (12.34–16.25)	11.84 ^b^ (10.59–13.36)	<0.001

n: number of animals. ^a, b^: The difference between measurements carrying different letters in the same row is statistically significant. The data is provided as median and minimum–maximum values.

**Table 7:** ROC analysis values of adiponectin, resistin, and omentin-1.

Parameters	AUC (95% CI)	*p* Value	Cut-Off	Sensitivity	Specificity	True Positive	True Negative
Adiponectin (ng/mL)	0.953 (0.891–1.000)	0.001	16.43	90%	87%	29/32	7/8
Resistin (ng/mL)	0.746 (0.601–0.892)	0.033	23.26	75%	75%	24/32	6/8
Omentin-1 (ng/mL)	0.969 (0.918–1.000)	0.001	12.97	87%	87%	28/32	7/8

AUC: area under the curve, CI: confidence interval.

**Table 8:** Statistical correlations between HCT, HGB, RBC, and adipose tissue hormones.

Parameters	HCT (%)	HGB (g/dL)	RBC (×10^12^/L)	Adiponectin (ng/mL)	Leptin (ng/mL)	Resistin (ng/mL)	Omentin-1 (ng/mL)
HCT (%)	1.000	0.733 **	0.863 **	−0.397 *	−0.034	−0.421 **	−0.328 *
HGB (g/dL)		1.000	0.736 **	−0.216	0.171	−0.288	−0.215
RBC (×10^12^/L)			1000	−0.338 *	0.167	−0.249	−0.231
Adiponectin (ng/mL)				1.000	0.479 **	0.430 **	0.614 **
Leptin (ng/mL)					1.000	0.440 **	0.515 **
Resistin (ng/mL)						1.000	0.341 *
Omentin-1 (ng/mL)							1.000

Spearman correlation * *p* < 0.05 and ** *p* < 0.01. HCT: hematocrit value, HGB: hemoglobin concentration, RBC: erythrocyte count.

## Data Availability

The original contributions presented in this study are included in the article. Further inquiries can be directed to the corresponding author.
